# Correction: Prediction of the ectasia screening index from raw Casia2 volume data for keratoconus identification by using convolutional neural networks

**DOI:** 10.1371/journal.pone.0338609

**Published:** 2025-12-09

**Authors:** Maziar Mirsalehi, Benjamin Fassbind, Andreas Streich, Achim Langenbucher

The Data Availability statement for this article is incorrect. The correct statement is: The dataset and codes have been uploaded to a repository. The DOI is: https://doi.org/10.5281/zenodo.16884052.

## References

[1] Mirsalehi M, Fassbind B, Streich A, Langenbucher A. Prediction of the ectasia screening index from raw Casia2 volume data for keratoconus identification by using convolutional neural networks. PLoS One. 2025;20(9):e0311036. doi: 10.1371/journal.pone.0311036 40892937 PMC12404543

