# Correction: Prediction of the ectasia screening index from raw Casia2 volume data for keratoconus identification by using convolutional neural networks

**DOI:** 10.1371/journal.pone.0351460

**Published:** 2026-06-09

**Authors:** Maziar Mirsalehi, Benjamin Fassbind, Andreas Streich, Achim Langenbucher

In the Results subsection of the Abstract, there is an error in the last sentence of the paragraph. The correct sentence is: In the classification task, the three networks yielded an accuracy of 94.81%, 95.27% and 95.83%, respectively; a sensitivity of 92.08%, 94.64% and 94.17%, respectively; a specificity of 96.61%, 95.69% and 96.92%, respectively; a positive predictive value of 94.72%, 93.55% and 95.28%, respectively; and a F1 score of 93.38%, 94.09% and 94.73%, respectively.

In Table 4, the accuracy and sensitivity value for adapted ResNet 18, and F1 score value for adapted EfficientNetB0 are incorrect. Please see the correct Table 4 here.

**Table 4:** Evaluation metrics for CNN architectures. *Abbreviations:* CNN = Convolutional Neural Network, PPV = Positive Predictive Value.

CNN architecture	Metrics				
	Accuracy	Sensitivity	Specificity	PPV	F1 Score
adapted ResNet18	0.9481	0.9208	0.9661	0.9472	0.9338
adapted DenseNet121	0.9527	0.9464	0.9569	0.9355	0.9409
**adapted EfficientNetB0**	**0.9583**	**0.9417**	**0.9692**	**0.9528**	**0.9473**
CorNet [5]	0.9213	0.9249	0.9154	0.9477	0.9362
KerNet [6]	0.9474	0.9371	None	0.9410	0.9389
CorNeXt [8]	0.9256	0.8407	1	None	0.9134
